# Supplementary material for: Effects of Short-Term Treatment with α-Lipoic Acid on Neuropathic Pain and Biomarkers of DNA Damage in Patients with Diabetes Mellitus
Source: Pharmaceuticals (Basel). 2024 Nov 16;17(11):1538. doi: 10.3390/ph17111538 (PMC11597811; doi:10.3390/ph17111538)
Supplement: Supplementary file 1 [file pharmaceuticals-17-01538-s001.zip › Supplementary Table S1.pdf]

Supplementary Table S1. Individual data of subjects with diabetes

Patient No.	Diag-nosis	Age, years	Gen-der	BMI, kg/m^2	HbA1c, %	Creati-nine, µmol/L	eGFR, mL/min/ 1.73m^2	Number of chronic co-morbidities	Treatment with ALA (mg x days)	Pain intensity		SCE/cell		MN, ‰		8-OHdG, ng/ml	
										Before	After	Before	After	Before	After	Before	After
1	T1DM	23	M	20.9	>7*	62	134	0	600 x 6	0	0	8.44	8.06	1.79	2.14	12.30	n.d.
2	T1DM	56	F	29.0	8.8	63	95	4	600 x 5	8	2	13.18	10.22	8.03	6.81	23.34	n.d.
3	T1DM	37	F	20.0	7.2	61	112	0	600 x 5	8	3	9.42	8.16	5.01	n.d.	11.78	n.d.
4	T1DM	49	M	20.8	11.3	60	113	2	600 x 5	0	0	7.82	9.34	3.33	4.17	12.35	9.11
5	T1DM	43	F	21.5	>7*	55	111	1	600 x 5	4	3	9.20	11.42	5.39	3.61	13.67	10.88
6	T1DM	25	M	25.3	6.2	100	90	0	600 x 9	4	2	7.40	7.34	n.d.	n.d.	10.01	8.63
7	T2DM	61	F	42.4	8.9	41	108	2	600 x 5	8	5	10.74	8.48	4.35	4.69	n.d.	n.d.
8	T2DM	48	M	29.6	11.4	78	101	2	600 x 5	7	4	10.38	10.94	4.02	5.35	n.d.	n.d.
9	T2DM	69	M	29.7	8	83	82	5	600 x 9	6	3	10.52	8.58	2.73	2.63	10.46	n.d.
10	T2DM	53	M	32.9	7.3	77	99	2	600 x 4	10	5	9.88	9.58	2.55	2.56	11.90	n.d.
11	T2DM	60	F	34.2	8	77	73	4	600 x 4	10	5	8.90	8.06	6.16	4.20	13.21	9.93
12	T2DM	61	M	28.9	12.6	82	94	2	600 x 4	3	3	8.80	8.82	3.30	2.78	9.85	7.63
13	T2DM	60	F	36.7	<7*	78	71	4	600 x 5	6	4	10.2	11.72	3.21	2.62	20.73	16.63
14	T2DM	81	F	31.2	7.2	62	82	5	600 x 7	7	6	8.76	7.42	5.07	4.87	11.52	10.54
15	T2DM	66	F	33.6	8.5	67	86	2	600 x 6	0	0	7.92	9.24	4.92	5.84	14.06	12.61
16	T2DM	60	F	39.2	9.2	54	103	3	600 x 6	6	0	8.48	7.92	4.11	4.97	13.67	13.04
Mean		53.2								6.69**	3.46**	9.38	9.08	4.26	4.09	13.49	11.00
SD		15.4								2.17	1.61	1.43	1.37	1.58	1.42	3.89	2.75

*Defined by the anamnesis

** Patients 1, 4 and 15 (with initial 0 values) not included

T1DM=type 1 diabetes mellitus; T2DM=type 2 diabetes mellitus; F=female; M=male; BMI=body mass index; HbA1c=glycated haemoglobin; eGFR=estimated glomerular filtration rate; ALA=α-lipoic acid; SCE=sister-chromatid exchange; MN=micronucleated cell (lymphocyte); 8-OHdG=8-oxo-2’-deoxyguanosine; n.d.=no data.

Number of chronic comorbidities was determined exactly as in: Chima, C.C.; Salemi, J.L.; Wang, M.; Mejia De Grubb, M.C.; Gonzalez, S.J.; Zoorob, R.J. Multimorbidity Is Associated with Increased Rates of Depression in Patients Hospitalized with Diabetes Mellitus in the United States. *Journal of Diabetes and its Complications* **2017**, *31*, 1571–1579, doi:10.1016/j.jdiacomp.2017.08.001.
