# Supplementary material for: Effects of Short-Term Treatment with α-Lipoic Acid on Neuropathic Pain and Biomarkers of DNA Damage in Patients with Diabetes Mellitus
Source: Pharmaceuticals (Basel). 2024 Nov 16;17(11):1538. doi: 10.3390/ph17111538 (PMC11597811; doi:10.3390/ph17111538)
Supplement: Supplementary file 1 [file pharmaceuticals-17-01538-s001.zip › Supplementary Table S2.pdf]

Supplementary Table S2. Individual data of non-diabetic subjects

Patient No.	Age, years	Gender	BMI, kg/m^2	Fasting glycemia, mmol/l	Number of chronic co-morbidities	SCE/cell	MN, ‰	8-OHdG, ng/ml
1	32	F	20.6	5.2	0	7.28	2.95	8.05
2	32	M	26.5	4.5	0	9.20	1.27	n.d.
3	36	M	23.0	4.5	0	6.32	2.18	14.02
4	38	M	26.3	4.9	0	10.42	1.81	13.10
5	41	F	23.5	4.5	0	7.86	3.72	11.36
6	41	M	28.9	5.7	0	6.88	4.06	14.86
7	41	F	25.6	4.8	0	7.72	6.64	12.51
8	41	F	20.6	4.8	0	8.68	5.61	8.34
9	41	F	28.1	4.6	0	6.58	6.73	9.66
10	41	F	20.0	5.3	0	8.28	2.18	n.d.
11	43	M	27.4	5.4	0	8.62	2.51	14.65
12	45	F	34.0	5.5	0	4.92	2.19	10.01
13	53	F	30.5	5.8	0	5.62	5.06	13.27
14	56	M	28.3	6.0	0	8.50	4.59	7.29
15	62	F	29.8	5.3	2	10.10	4.82	11.01
16	70	F	34.3	5.1	2	8.10	4.23	17.13
Mean	***44.6***					***7.82***	***3.78***	***11.80***
SD	***10.5***					***1.51***	***1.71***	***2.91***

F=female; M=male; BMI=body mass index; SCE=sister-chromatid exchange; MN=micronucleated cell (lymphocyte); 8-OHdG=8-oxo-2'-deoxyguanosine; n.d.=no data.

Number of chronic comorbidities was determined exactly as in: Chima, C.C.; Salemi, J.L.; Wang, M.; Mejia De Grubb, M.C.; Gonzalez, S.J.; Zoorob, R.J. Multimorbidity Is Associated with Increased Rates of Depression in Patients Hospitalized with Diabetes Mellitus in the United States. *Journal of Diabetes and its Complications* **2017**, *31*, 1571–1579, doi:10.1016/j.jdiacomp.2017.08.001.
